# Supplementary material for: Fabrication and Study of Dextran/Sulfonated Polysulfone Blend Membranes for Low-Density Lipoprotein Adsorption
Source: Materials (Basel). 2023 Jun 27;16(13):4641. doi: 10.3390/ma16134641 (PMC10342430; doi:10.3390/ma16134641)
Supplement: Supplementary file 1 [file materials-16-04641-s001.zip › materials-2436428-supplementary.pdf]

materials

MDPI

Supplementary Materials

Fabrication and Study of dextran/sulfonated polysulfone blend membranes for low-density lipoprotein adsorption

Fei Fang [1,2], Hai-Yang Zhao [2], Rui Wang [2], Qi Chen [2], Qiong-Yan Wang [2,*] and Qing-Hua Zhang [1,*]

[1] College of Chemical and Biological Engineering, Zhejiang University, Hangzhou, China, 310027
[2] Research and Development Center, Zhejiang Sucon Silicone Co., Ltd., Shaoxing 312088, China;
* Correspondence: wqy2040@163.com (Q.-Y.W.); qhzhang@zju.edu.cn (Q.-H.Z.)

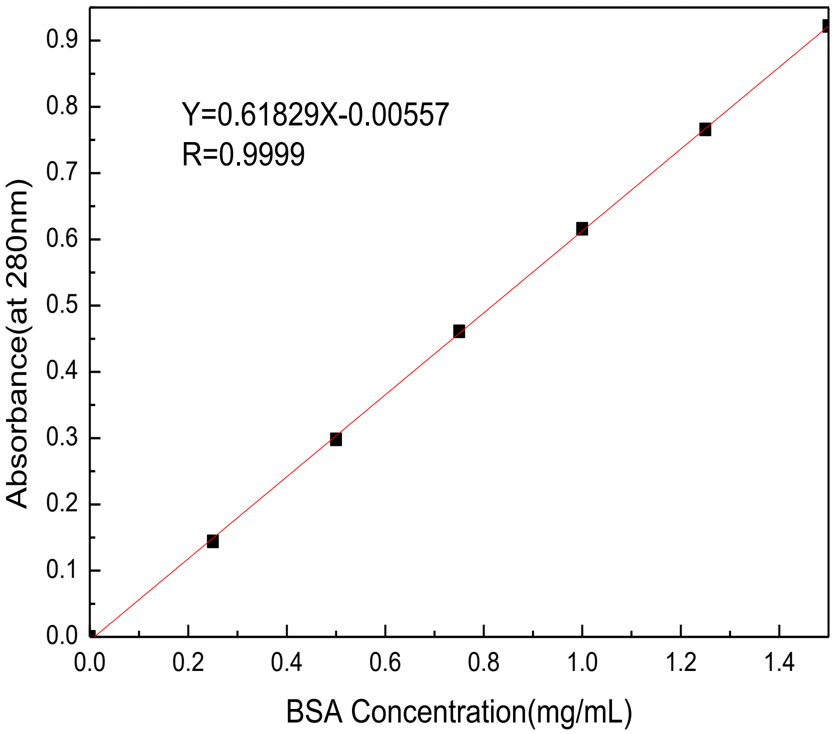

Figure S1. Standard curves of the BSA adsorption.

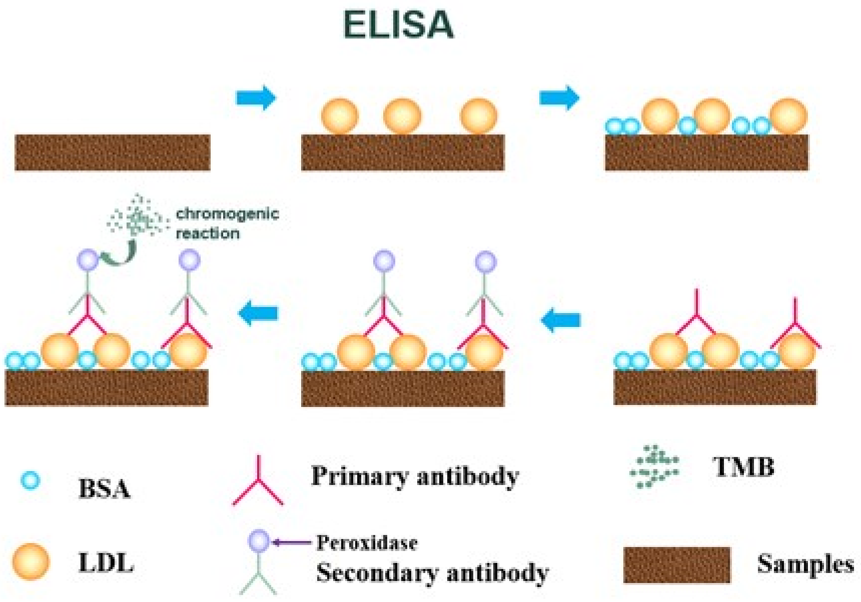

Figure S2. Standard curves of the BSA adsorption.
